# Novel Bioinformatics Approach Identifies Transcriptional Profiles of Lineage-Specific Transposable Elements at Distinct Loci in the Human Dorsolateral Prefrontal Cortex

**DOI:** 10.1093/molbev/msy143

**Published:** 2018-07-20

**Authors:** Guia Guffanti, Andrew Bartlett, Torsten Klengel, Claudia Klengel, Richard Hunter, Gennadi Glinsky, Fabio Macciardi

**Affiliations:** 1 Department of Psychiatry, Harvard Medical School, Cambridge, MA; 2 Division of Depression and Anxiety, McLean Hospital, Belmont, MA; 3 Department of Psychology, University of Massachusetts, Boston, MA; 4 Department of Psychiatry and Psychotherapy, University Medical Center Göttingen, Georg-August-University, Goettingen, Germany; 5 Translational & Functional Genomics, Institute of Engineering in Medicine, University of California San Diego, La Jolla, CA; 6 Department of Psychiatry and Human Behavior, University of California Irvine, Irvine, CA

**Keywords:** transposable elements, dorsolateral prefrontal cortex, comparative genomics, RNA-mediated epigenetics and RNA-seq, schizophrenia, transcription factor binding sites

## Abstract

Expression of transposable elements (TE) is transiently activated during human preimplantation embryogenesis in a developmental stage- and cell type-specific manner and TE-mediated epigenetic regulation is intrinsically wired in developmental genetic networks in human embryos and embryonic stem cells. However, there are no systematic studies devoted to a comprehensive analysis of the TE transcriptome in human adult organs and tissues, including human neural tissues. To investigate TE expression in the human Dorsolateral Prefrontal Cortex (DLPFC), we developed and validated a straightforward analytical approach to chart quantitative genome-wide expression profiles of all annotated TE loci based on unambiguous mapping of discrete TE-encoded transcripts using a de novo assembly strategy. To initially evaluate the potential regulatory impact of DLPFC-expressed TE, we adopted a comparative evolutionary genomics approach across humans, primates, and rodents to document conservation patterns, lineage-specificity, and colocalizations with transcription factor binding sites mapped within primate- and human-specific TE. We identified 654,665 transcripts expressed from 477,507 distinct loci of different TE classes and families, the majority of which appear to have originated from primate-specific sequences. We discovered 4,687 human-specific and transcriptionally active TEs in DLPFC, of which the prominent majority (80.2%) appears spliced. Our analyses revealed significant associations of DLPFC-expressed TE with primate- and human-specific transcription factor binding sites, suggesting potential cross-talks of concordant regulatory functions. We identified 1,689 TEs differentially expressed in the DLPFC of Schizophrenia patients, a majority of which is located within introns of 1,137 protein-coding genes. Our findings imply that identified DLPFC-expressed TEs may affect human brain structures and functions following different evolutionary trajectories. On one side, hundreds of thousands of TEs maintained a remarkably high conservation for ∼8 My of primates’ evolution, suggesting that they are likely conveying evolutionary-constrained primate-specific regulatory functions. In parallel, thousands of transcriptionally active human-specific TE loci emerged more recently, suggesting that they could be relevant for human-specific behavioral or cognitive functions.

## Introduction

The regulatory, noncoding DNA makes up ∼98% of the human genome and plays a fundamental role in the evolution and development of the nervous system (Harpending et al. 1998; Cordaux and Batzer 2009; Hormozdiari et al. 2013; Thakurela et al. 2015; Villar et al. 2015; Berto et al. 2016; van Gestel and Weissing 2016; Vermunt et al. 2016). About half of the noncoding regulatory genome consists of retrotransposons, a large group of transposable elements (TEs) that can “copy and paste” their own DNA in the host genome (de Koning et al. 2011). Although the vast majority of TEs in the human genome are no longer transpositionally active, they can still be functionally relevant as exapted enhancers and transcription start sites (either HERVs, Human Endogenous Retro Viruses, LINE1s, Long INterspersed Elements and Alus) (Rangwala et al. 2009; Deininger 2011; Su et al. 2014; Babaian and Mager 2016), by inserting Transcription Factor Binding Sites (TFBS) (Emera and Wagner 2012; Lynch et al. 2015) or even introducing novel RNA genes such as long noncoding RNAs (lncRNAs) (Hezroni et al. 2015). These proposed functional roles suggest that TEs are essential elements in defining the regulatory and structural features of the human genome (Goodier and Kazazian 2008; Rangwala et al. 2009; Huang et al. 2010; Poduri et al. 2013; Guffanti et al. 2014; Elbarbary et al. 2016; Mallona et al. 2016; Mita and Boeke 2016; Chen and Yang 2017). Evidence is growing that TE-mediated epigenetic regulation, which belongs to the broad category of RNA-mediated epigenetic regulatory mechanisms of gene expression (Holoch and Moazed 2015), is indeed a key process to organize developmental gene-network in human embryonic (hESC) and induced pluripotent (hiPSC) stem cells and that TEs may rewire differentiation and cell fate-defining gene-networks (Pollard et al. 2006; Cowley and Oakey 2013; Jacques et al. 2013; Franchini and Pollard 2015a, 2015b; Lynch et al. 2015). Very recent findings show that functional enrichment of OCT4 and NANOG characterizes hi-activity enhancers on both naïve and primed hESC, a condition that relates to stem cells maintenance with a complex and not yet defined cell-type specificity (and chromatin segmentation) (de Wit et al. 2013; Barakat et al. 2018). Suppression of NANOG expression induces neural differentiation (Chambers et al. 2009) with the spatiotemporal regulation of gene expression requiring a complex concerted action of many more TFs than purely OCT4 and NANOG in both hESC and iPSC (Theunissen et al. 2016; Theunissen and Jaenisch 2017). Recent analyses (Glinsky 2015, 2018) have already shown that a significant proportion of primate-specific TEs, notably LTR7/HERV-H, LTR5-Hs, and L1Hs/L1PA2, harbor 99.8% of the candidate primate- and human-specific regulatory loci (PHSRL) with putative TFBS in the genome of human embryonic stem cells (hESC). These candidate PHSRL display selective and site-specific binding of critical developmental and stem cell fate regulators (*NANOG* [Nanoghomeobox], *POU5F1* [POUclass5homeobox1], CCCT C-binding factor [*CTCF*], Lamin B1[LMNB1]) and are preferentially located within the matrix of transcriptionally active DNA segments that are hypermethylated in hESC. Candidate human-specific NANOG-binding sites are enriched near protein-coding genes regulating brain size, pluripotency lncRNAs, hESC enhancers, and 5-hydroxymethylcytosine-harboring regions immediately adjacent to binding sites (Kunarso et al. 2010; Guttman et al. 2011; Macia et al. 2011; Jacques et al. 2013; Kapusta et al. 2013). We also previously identified in silico thousands of regulatory sequences that are either highly conserved across primate evolution and evolved by the exaptation of highly conserved ancestral DNA or were driven by the species-specific insertions of TEs in the human lineage (Glinsky 2016).

Despite these significant findings, and some initial experimental results that revealed the putative regulatory role of TEs in the neural genome (Coufal et al. 2009; Bundo et al. 2014; Erwin et al. 2014; Su et al. 2014; Zhang et al. 2015; Guffanti et al. 2016; Doyle et al. 2017; Sur et al. 2017), we are still lacking a detailed and comprehensive knowledge of such a TE-controlled regulation. A better understanding of TEs’ regulation will also contribute to appreciate the relative importance of retrotransposition events in germ-line and in somatically differentiated cells (Baillie et al. 2011; Evrony et al. 2012; Fasching et al. 2015; McConnell et al. 2017). Moreover, variations in DNA sequences and RNA expression of noncoding regulatory elements, rather than protein-coding genes, have also been implicated as major risk factors in neuropsychiatric disorders, like schizophrenia (Roussos et al. 2014; Srinivasan et al. 2015; Xu et al. 2015). These evidences suggest that a better knowledge of how TEs control developmental programs and cellular reprogramming is essential to design targeted therapeutic approaches in schizophrenia (Brami-Cherrier et al. 2014a, 2014b) and other neuropsychiatric disorders. Therefore, the systematic exploration of TE-mediated epigenetic programs in the neural tissues is becoming a critically important step in our efforts to reveal their role in the evolution and development of cognitive functions.

Many TEs are expressed very early at specific developmental periods, beginning with the early stages of the human preimplantation embryogenesis (Faulkner et al. 2009; Faulkner 2013; Fort et al. 2014; Glinsky 2015, 2018; Gerdes et al. 2016; Glinsky 2016), and essentially contribute to regulate primary developmental gene networks (Fort et al. 2014; Lu et al. 2014; Durruthy-Durruthy et al. 2016; Theunissen et al. 2016; Kobayashi et al. 2017; Theunissen and Jaenisch 2017). Functionally active TEs have been described and classified as lncRNAs, enhancers, insulators, or promoters of neighboring genes in various tissues with a putative functional role in neuropsychiatric disorders, like schizophrenia (Subramanian et al. 2011; Perron, Germi, et al. 2012; Perron, Hamdani, et al. 2012; Hegyi 2013; Suntsova et al. 2013; Guffanti et al. 2014). However, almost all studies on TE expression lumped together all the elements of given subfamilies, like the analysis of HERV-K expression in schizophrenia (Yolken et al. 2000; Frank et al. 2005), rather than pinpointing the exact genomic coordinates of specific transcriptionally active TE loci. This lack of genomic-locus-level resolution severely limited our ability to understand the potential regulatory implications of activated TE expression and to assess the magnitude of a putative functional impact.

Recent RNA-seq studies revealed a widespread pattern of expression of different HERV families in different cell lines and tissues, both in health and diseases, and demonstrated the feasibility of unambiguously profiling individual HERV loci at their specific chromosomal locations (Agoni et al. 2013; Suntsova et al. 2013, 2015; Criscione et al. 2014; Haase et al. 2015). While many genome-wide transcriptome studies focused on HERVs and their putative role as enhancers (Arner et al. 2015; Reilly et al. 2015; Chuong et al. 2016; Gerdes et al. 2016; Vargiu et al. 2016), compelling experimental evidences are emerging documenting both the transcriptional activity of L1Hs in human somatic tissues in addition to their well-established ability of insertional mutagenesis, suggesting a potential role also for L1s as regulatory elements (Belancio et al. 2010; Philippe et al. 2016; Deininger et al. 2017; Macia et al. 2017) and Alus as enhancers (Su et al. 2014; Schneider et al. 2018). Overall, these studies suggest a potential role for diverse families of TEs as regulatory elements of transcriptional dynamics. They indicate that expression of both LTR and non-LTR TE loci can be accurately measured and their regulatory effect on neighboring or distant genes can be experimentally assessed, although the repetitive nature and the complex evolutionary history of TEs make it difficult to precisely map and quantify the degree of expression at discrete TE loci.

To address these challenges, we developed and validated a straightforward analytical strategy to obtain the unambiguous identification of the quantitative expression signatures of discrete TE loci on a genome-wide scale using a de novo assembly methodology tailored to explore the human TE transcriptome. Then, we adopted a comparative evolutionary genomics approach across human, primates, and rodents to identify conservation and lineage-specificity, of transcriptionally active TEs. Comparative expression profiling analysis provides an important first step to assess the function of regulatory elements such as TEs. Moreover, the availability of a comprehensive genome-wide annotated catalogue of TEs expressed in the human DLPFC has the potential to generate testable hypotheses to evaluate the regulatory role of TEs in shaping the development of human neural tissue and the evolution of our unique cognitive functions. To our knowledge, this is the first report of a successful genome-wide mapping of actively transcribed individual TE derived candidate PHSRL in human postmortem DLPFC tissues.

## Results

### Analysis of TE Transcription in Human DLPFC

**Fig. 1.**
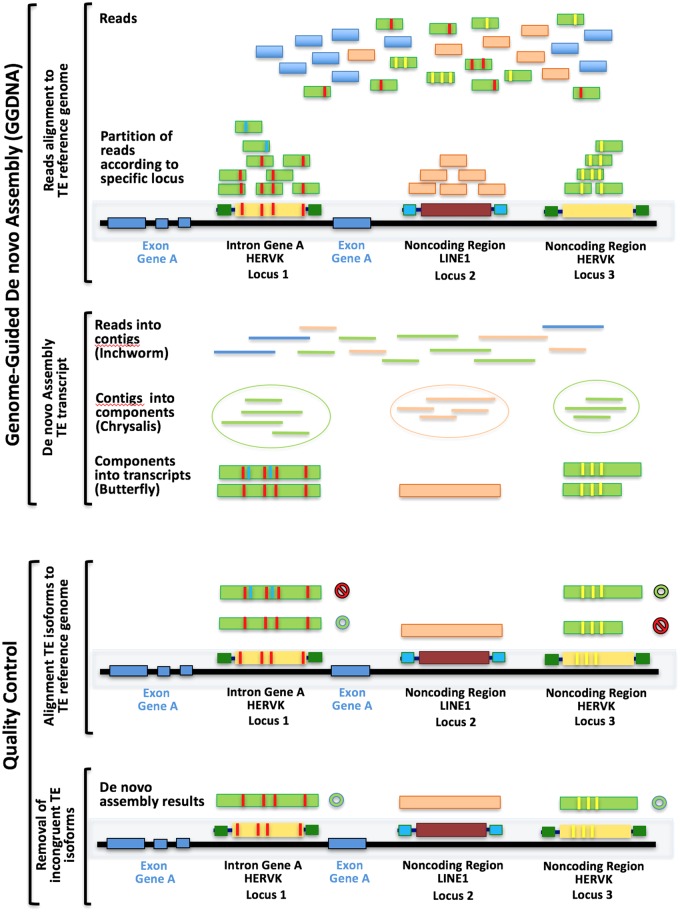
A graphical representation of the GGDNA workflow used to identify each single expressed TE transcript from RNA-seq data of DLPFC in our sample. The reads generated by the RNA-seq procedures are first aligned to the annotated reference TE database from Repeatmasker, then reads at each single locus are assembled de novo. Transcripts with <95% sequence identity with the reference and/or align at <90% of their length are discarded during the step of quality control: reads that are discarded are identified with the symbol 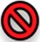 and reads that are carried on with the symbol 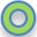. See also text for details of the procedures.

We developed a transcriptome assembly/annotation pipeline that we used to process raw RNA sequencing data with a genome-guided de novo assembly workflow adapted to detect the transcriptional profiles of TEs. Figure 1 shows how this bioinformatics pipeline implements a robust method for the de novo reconstruction of transcripts from RNA-seq data, based on the Trinity genome-guided de novo assembly (GGDNA).

We applied GGDNA to more than two billion (10^9^) RNA-sequencing reads from 19 individual DLPFC samples. As preliminary quality control procedure, we removed all reads with mean quality <20 using FastQC (see Materials and Methods). The application of GGDNA yielded a set of candidate TE transcripts that aligned to the reference sequences of 1,766,735 discrete TEs reported in Repeatmasker. Because individual TE transcripts could align with more than one reference TE locus, we implemented a sequence alignment strategy designed to univocally identify discrete TE-encoded transcripts that are stringently aligned to their unique genomic locations. To reach this goal, we imposed that TE transcripts i) must align with a TE reference sequence for at least 90% of the transcript length, which reduced the possible alignments to 1,675,434 TE loci (96.4%); and ii) must display at least 95% identity between the sequences of each candidate TE-derived transcript and the matched reference TE sequence from RepBase/Repeatmasker, which further reduced the number of alignments to 1,239,821 (70.2%) TE loci.

We then screened this set of candidate TE transcripts and removed sequences that were still mapping with identical parameters to more than one genomic location, by iii) retaining only those TE transcripts that display 100% identity sequence with the corresponding reference TEs. This high-stringency sequence selection resulted in the identification of 657,062 TE transcripts that we considered our best “primary alignment” for TEs in our DLPFC samples. From this set, we removed 2,397 additional manually curated transcripts (0.36% of 675,062) for which their sequences failed to unambiguously align to a single genomic TE locus and mapped to more than one locus with similar alignment/identity (100%) and quality scores. This quality control filtering protocol yielded a total of 654,665 (37%) individual transcripts that mapped only once to the hg38 human genome. Detailed descriptions of the findings and associate statistical analyses are reported in the text and also in tables 1–3 and Additional Files 1–4. Each transcript was assigned the genomic coordinates of the primary alignment locus, corresponding to a total of 477,507 reference TE loci. We obtained an average of 349 (± 185.4) reads per transcript, with only the 0.002% of transcripts supported by ten or less than ten reads (table 1).

**Table 1.** Distinct Classes of Primate- and Human-Specific TE Loci Transcriptionally Active in the Dorsolateral Prefrontal Cortex (DLPFC) of Human Brain.

Classification Category	TE Transcripts Expressed in DLPFC	Primate-Specific TEs, *n* (%)	Human-Specific TEs, *n* (%)	Average Number of Reads per Transcript
LTR class	126,849	101,733 (80.2%)	596 (0.47%)	303
LINE class	319,509	245,383 (76.8%)	2,108 (0.66%)	468
SINE class	155,366	132,216 (85.1%)	715 (0.46%)	117
DNA class	43,608	31,965 (73.3%)	87 (0.19%)	286
Other (SVA)	3,317	3,313 (99.9%)	770 (23.2%)	400
Total	654,665	519,804 (79.4%)	4,276 (0.66%)	346

Note.—The majority of the transcripts (94.1%) are supported by >20 reads (88.7% by 20–1,000 reads and 5.7% by >1,000 reads), and only 5.9% by <20 reads. TE loci that have <10% of bases remapped during the conversion from the human genome (hg38) to the mouse genome (mm10) were defined as primate-specific loci; TE loci that have <10% of bases remapped during the conversion to both Chimpanzee (PanTro5) and Bonobo genomes were defined as human-specific loci; TE, transposable elements.

**Table 2.** Primate- and Human-Specific TE Transcripts Originated from Loci Harboring Binding Sites of the Master Pluripotency Regulators NANOG, POU5F1, and CTCF.

Classification Category	Primate-Specific Loci, *n* (%)	*P* Values*	Nonhuman Primates’ Loci, *n* (%)	*P* Values*	Human-Specific Loci, *n* (%)	*P* Values*
NANOG-binding sites						
Genome (hg38)	29,083		28,267		816	
Expected number of expressed loci	5,172		5,171		71	
Observed number of expressed loci in postmortem DLPFC samples	6,399 (22%)	3.37E-37	6,197 (21.9%)	5.24E-27	202 (24.8%)	1.79E-18
CTCF-binding sites						
Genome (hg38)	28,236		27,661		575	
Expected number of expressed loci	5,021		5,060		50	
Observed number of expressed loci in postmortem DLPFC samples	4,144 (14.7%)	1.47E-23	4,113 (14.9%)	2.70E-27	31 (5.4%)	0.037
OCT4/POU5F1-binding sites						
Genome (hg38)	12,458		10,130		2,328	
Expected number of expressed loci	2,216		1,853		203	
Observed number of expressed loci in postmortem DLPFC samples	1,866 (15%)	2.28E-09	1,774 (17.5%)	0.15	92 (4%)	2.21E-11
NANOG + POU5F1 + CTCF binding sites						
Genome (hg38)	69,777		66,058		3719	
Observed number of expressed loci in postmortem DLPFC samples	12,409 (17.8%)		12,084 (18.3%)		325 (8.7%)	

* *P* values reflecting the statistical significance between the observed and expected numbers of expressed loci was estimated using a two-tailed Fisher’s exact test; the Expected numbers of expressed loci were calculated based on the percentage of all expressed TE-derived loci in the corresponding classification category; Nonhuman primates’ loci refer to conserved in primates loci common to humans and nonhuman primates.

**Table 3.** Two Distinct Evolutionary Patterns of Highly Conserved in Primates and Human-Specific TE Loci Transcriptionally Active in Human’s DLPFC.

TE Family	DLPFC Expressed RNAs (*n*)	DLPFC Expressed Loci (*n*)	Highly Conserved in Primates Loci, *n* (%)	Human-Specific Loci, *n* (%)	Humans/Primates Ratio	Highly Conserved and Human-Specific Loci (*n*)	Highly Conserved and Human-Specific Loci (%)
L1Hs	1,240	463	51 (11%)	354 (76.5%)	6.9	405	87.5
L1PA2	4,244	1,474	154 (10.4%)	688 (46.7%)	4.5	842	57.1
SVA	3,317	1,560	54 (3.5%)	841 (53.9%)	15.6	895	57.4
*Human-specific*	*8,801*	*3,497*	*259 (7.4%)*	*1883 (53.8%)*	*7.3*	*2,142*	*61.3*
LTR5	854	476	302 (63.4%)	66 (13.9%)	−4.6	368	77.3
HERVK	1,447	563	434 (77.1%)	49 (8.7%)	−8.9	483	85.8
HERV9	483	172	140 (81.4%)	10 (5.8%)	−14	150	87.2
HERV (various)	4,293	1,925	533 (89.4%)	13 (2.2%)	−41	546	91.6
LTR7	832	634	507 (80%)	14 (2.2%)	−36.2	521	82.2
HERVH	2,365	1,101	855 (77.7%)	30 (2.7%)	−28.5	886	80.4
AluY	14,288	12,184	8605 (70.6%)	399 (3.3%)	−21.6	9,004	73.9
*Highly conserved in primates*	*24,852*	*17,055*	*11,376 (72.3%)*	*581*	*−19.5*	*11,957*	*76*

Note.—TE loci that have at least 95% of bases remapped during the direct and reciprocal conversions to the genomes of humans (hg38), Chimpanzee (PanTro5), and Bonobo were defined as highly conserved in primate sequences; TE loci that have <10% of bases remapped during the conversion from the human genome (hg38) to both Chimpanzee (PanTro5) and Bonobo genomes were defined as human-specific loci. Values in italic font report the cumulative numbers for corresponding classification categories.

### Genomic Location of TE Loci Relative to Protein-Coding Genes and Noncoding RNA Transcripts

The sizes of the transcriptionally active TEs in our set of postmortem DLPFC samples ranged from 224 to 8,462 nucleotides (with mean size of 396 and median of 314 nucleotides), suggesting that they represent distinct classes of RNAs with putatively diverse biological functions. Three classes (LINE, 48.5%; SINE, 24.2%; LTR, 19.3%) and nine families of TEs were mostly represented among the DLPFC-expressed TEs, collectively encompassing 601,724 (92%) transcripts (table 1). We found that 82.5% of the observed TEs (*n* = 540,099 transcripts) map within the boundaries of 14,255 protein-coding and 8,608 noncoding RNA genes. Notably, the great majority of these TEs map to noncoding regions (i.e., either introns, 5′ or 3′-UTR), although an intriguing proportion overlaps also with exons and a few with whole CoDing Sequences (CDSs). Since 63,100 of the 654,665 individual TE-derived transcripts map within the noncoding regions of more than one human gene due to the overlap of multiple genes within the same annotated chromosomal regions, we have a total of 717,765 transcribed loci if we consider this ambiguity in annotation (supplementary tables 1–4, Supplementary Material online, show the numbers of TEs mapped by our transcripts for each major TE class).

### Experimental Validation of TE Transcription at Specific Genomic Locations

To assess the robustness of our analytical pipeline, we performed a quantitative RT-PCR (qRT-PCR) validation of five HERVKC4 transcripts in 4 of the 19 available RNAs from the postmortem DLPFC samples.

**Fig. 2.**
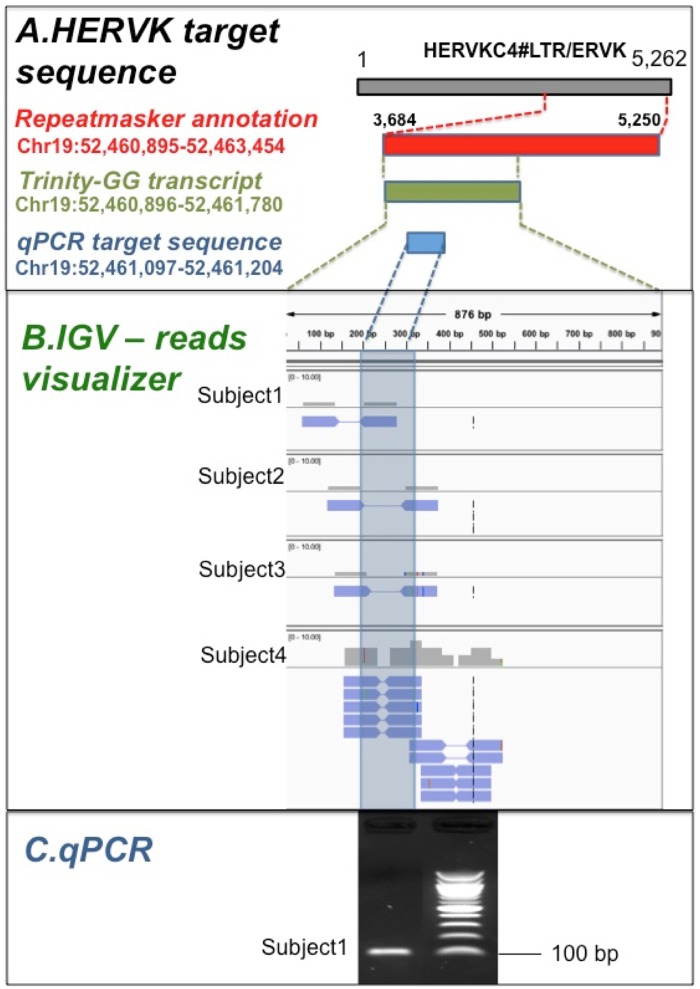
Validation of the actively transcribed HERV locus HERVKC4 on chromosome 19 in human DLPFC. A (top figure). The cartoon reports the sequence coordinates (not in scale) of the tested HERVKC4 (in red), the transcript assembled by GGDNA (in green) and the region captured by qPCR (in blue). B (middle figure). Visualization of the read alignment to the GGDNA transcript for each of the four RNA samples; highlighted in blue the region corresponding to the qPCR product. C (bottom figure). Result on agarose gel of the qPCR product for one RNA sample with the relative length size of 100 bp. Quantitative RT-PCR (qRT-PCR) validation experiments of four HERVKC4 transcripts were carried-out on four of the nineteen analyzed RNAs extracted from the human DLPFC samples (three controls and one Schizophrenia sample). The sequence identity of the purified PCR products has been confirmed by direct sequencing.

We initially looked at HERVKC4 transcripts, because HERVKC4 represents one of evolutionarily youngest types of HERVK retroviruses: this also means that HERVKC4 sequences at distinct genomic locations are likely to be highly similar from one instance to another due to the lesser evolutionary time elapsed to accumulate unique genetic mutations. To assess false positives arising from background levels of genomic DNA and protein coding mRNA or pre-mRNA (e.g., intronic mRNA incorrectly spliced), we carefully selected control reactions for the amplification of HERVKC4 sequences mapping to i) gene desert regions and ii) overlapping the intron of a protein coding mRNA but on the opposite strand. Figure 2 reports the details of the validation experiment of a single HERVKC4 mapping to chromosome 19 as an example.

To date, we have completed the successful validation of five HERVKC4 loci that are transcriptionally active in human DLPFC (two on chr1; two on chr19; and one on chr6). For each tested HERVKC4 locus, we generated more than one amplicon with a different set of primers (on an average, about four amplicons for each tested HERVKC4 locus), then we purified and sequenced the resulting qPCR products with Sanger sequencing. Blasting these sequences against the human reference genome, we observed 100% sequence identity with the predicted HERVKC4 loci located exactly where we expected it should have been based on the results of our computational mapping pipeline. Then, checking the results from RNA-seq with the qPCR for each individually tested loci, we verified that we observed the expected results only in samples from those subjects who had that specific instance of the HERVKC4 transcript expressed.

The example of the validation experiments reported in fig. 2 conclusively shows that i) we were able to blast the sequenced amplicon exactly in the precise genomic location that we were expecting (and within the sequence we obtained from RNA-seq that falls within the Repeatmasker sequence for that particular HERVKC4 locus); and that ii) the sequence of the RT-qPCR product align perfectly with the RNA-seq reads from all four subjects with at least one pair-read per DLPFC sample, thus unequivocally confirming the RT-qPCR product sequence identity to the results we obtained from RNA-seq data.

Then, we selected various types of class I TEs as additional targets for RT-qPCR validation experiments. Primers were designed against selected transcripts and run through NCBI BLAST against nr databases for *Homo sapiens* and *Rattus norvegicus* for confirmation of the predicted specificity of primers and amplicons. In addition, primers were built against the 18S ribosomal RNA and GAPDH gene. The 18S primers were built targeting the hyper-conserved domain with perfect amplicon sequence identity for both hg38 and rn6 18S reference sequences. Conversely, the GAPDH primers were built against the hg38 reference sequence and contain two mismatches per primer against the rn6 reference sequence. These housekeeping genes were used as positive internal controls for perfect specificity, 18S, and inefficient pseudospecificity, GAPDH. Given the truncation of many TEs, for large transcripts additional primer sets were designed targeting independent amplicons along the selected transcript to assess expression of the entire assembled transcript. All primers used have been reported in supplementary table 6, Supplementary Material online.

Amplification was observed in all cDNA samples for all targets except one: AluJo was the only target that apparently failed to amplify, as shown both by Ct data and verified by imaging of PCR product (supplementary table 7 and fig. 1, Supplementary Material online). For many targets (e.g., SVA_B; LTR5_HS, chr3; SVA_D; L1PA7 set2; L1M1 set2), rat gDNA showed no amplification indicating species specificity. For two targets (e.g., L1M1; L1PA7 set 2), significantly lower amplification was observed in rat gDNA reactions compared with human samples and PCR differences in melting temperature between samples and rat gDNA indicate differences in PCR product consistent with species specificity (supplementary tables 6 and 8, Supplementary Material online). For an additional subset of targets (e.g., L1Hs, chr2; L1PA7), low-level amplification was observed in rat gDNA reactions and similar melting temperatures for PCR products observed for both human samples and rat gDNA reactions. However, for these targets an in silico analysis revealed that the target amplicon was not found in the nr database for *rattus norvegicus* (supplementary table 6, Supplementary Material online)*.* Therefore, it seems reasonable to conclude that the low-level inefficient amplification observed for some targets in rat gDNA reflects nonspecific reactions and it is unlikely that it is due to amplification of the target amplicon.

We observed a greater than five-cycle difference (−6.62± 0.616 Ct: mean ±standard error for all validated TE targets) between the –RT sample and the reverse transcribed cDNA samples for the majority of targets. Assuming 100% efficiency, this equivalently suggests that only up to 3.1% of the amplification observed in the reverse transcribed cDNA samples can be attributed to residual genomic content. Therefore, we conclude that amplification of TE targets observed in the reverse transcribed samples is largely driven by RNA molecules. It cannot be explained by residual DNA contaminations of these repetitive elements in our samples and that the contribution of residual genomic content, if any, to cDNA amplification is sufficiently negligible. Collectively, these results validate the efficiency of our bioinformatics pipeline to correctly assess transcription in human postmortem DLPFC samples from a single discrete TE locus.

### TEs Harbor Human-Specific Loci with Putative Transcription Factor Binding Sites

Having developed a method to define high-quality TE transcriptional profiles in the human DLPFC, we sought to better characterize them using a comparative genomics approach. To enable a comparative evolutionary analysis of TEs that are actively transcribed in the postmortem DLPFC of our samples, we first identified primate- and human-specific TE loci expressed in DLPFC and then intersected the genomic coordinates of our TE transcripts with those derived from the primate- and human-specific TE loci harboring TFBS. We found that primate-specific (*n* = 564,314) and human-specific (*n* = 4,687) TEs are markedly overrepresented among all the expressed TEs in our samples (tables 1–3 and supplementary tables 1–4, Supplementary Material online). Table 1 reports the distribution of TE transcripts in the DLPFC by class and by evolutionary patterns of conservation, showing that 76.8% of all LINEs expressed in DLPFC are primate-specific, as well as 80.2% of all LTRs, 85.1% of all SINEs, and 99.9% of all SVAs. The relatively small number (*n* = 4,687) of candidate human-specific expressed TEs are mostly represented by L1Hs, L1PA2, SVA, and AluY sequences (supplementary tables 1–4, Supplementary Material online and fig. 2), the prominent majority (80.2%) of which appears spliced and was identified in our human DLPFC RNA-seq data set by segment fragments. Of these, 50.6% were identified by at least two segment fragments and 49.4% were identified by single segment fragments. The latter category appears spliced from the nascent RNA as supported by the evidence that the length of the transcript was < 90% of the length of the gDNA of the corresponding reference TE loci. The remaining group (20%) is represented by transcripts whose length almost entirely corresponded to the length defined by the coordinates of the gDNA of corresponding TE loci. As expected, this group consists of mostly Alu sequences, whose length ranges from 213 to 387 bp and for which there is no a consensus splicing model supported by experimental evidence (Deininger 2011; Pandey and Mukerji 2011; Lubelsky and Ulitsky 2018). These observations are consistent with the previous study reporting that TE-lncRNAs have greater splicing complexity compared with conventional lncRNAs defined by the exons/transcript and isoforms/gene ratios (Kelley and Rinn 2012).

A comprehensive genome-wide study of TE loci harboring TFBS in the human genome (Kunarso et al. 2010) identified 205,974 TFBS for the three master pluripotency regulators, namely, NANOG, OCT4 (POU5F1), and CTCF transcription factors. The follow-up report (Glinsky GV 2015) mapped these 205,974 TFBS across human, rodent, and primate reference genomes and identified 29,130, 14,003, and 29,018 primate-specific, and 826, 2,386, and 591 human-specific sequences at NANOG-, OCT4-, and CTCF-binding sites, respectively. We found that a significantly higher number of transcripts than expected by chance appears to derive from TE loci harboring TFBS for NANOG, OCT4 (POU5F1), and CTCF master pluripotency regulators for either primate-specific (*n* = 12,409; *P* < 1.00×10^−300^; hypergeometric test) or human-specific DLPFC-expressed TE loci (*n* = 325; *P* < 1.00^−300^; hypergeometric test). Comparing the relative prevalence of TFBS for NANOG, OCT4, and CTCF, we observed a significantly higher proportion of TE transcripts transcribed from loci harboring primate- and human-specific TFBS for NANOG than random (*P* = 3.37×10^−37^; and *P* = 1.79×10^−18^, respectively; hypergeometric test: table 2). In contrast, relatively smaller proportions of TEs harboring primate- and human-specific transcription factor-binding sites for OCT4 (*P* = 1.47×10^−23^; and *P* = 0.04, respectively; hypergeometric test) and CTCF (*P* = 2.28×10^−9^; and *P* = 2.21×10^−11^, respectively; hypergeometric test) were identified than expected by chance (table 2).

These findings are in agreement with the recent results of genome-wide proximity placement analyses of human-specific TFBS linking NANOG with gene expression regulatory networks of human fetal brain and adult neocortex (Glinsky 2017, 2018; Topalovic et al. 2017; Su et al. 2018). Collectively, these observations suggest that thousands of primate- and human-specific DLPFC-expressed TE loci that we have identified could likely have biologically significant functions.

### Evolutionary Dynamics of Highly Conserved-in-Primates and Human-Specific TE Loci Expressed in Human DLPFC

As expected, many transcripts aligning to L1Hs loci that are actively expressed in the DLPFC overlap with 246 truncated reference L1Hs (522 transcripts): of these, 124 transcripts may potentially represent L1 fragments incorporated into other cellular RNAs, being transcribed with the same strand orientation of protein-coding genes RNAs they appear to be part of (Deininger et al. 2017), while a meaningful interpretation of the origin of remaining transcripts (*n* = 398) is less evident. Some transcripts show a sequence similarity with L1-ORF1 (Moran et al. 1996; Kulpa and Moran 2005; Goodier et al. 2007; Sokolowski et al. 2017) and a few present ORF0-like sequences (Denli et al. 2015), making it clear that much work remains to be done to understand the possible functions of these transcripts, if any. However, we also detected the expression of 214 intact, full-length L1Hs loci, characterized by 6,032 or more nucleotides. The active transcription of these full-length L1Hs sequences is also consistent with their retained potential for transpositional activity.

We have also quantified the expression of 140,399 Alu transcripts, mostly represented by AluSx and AluY elements that are the evolutionarily youngest subfamilies of the human SINEs. While all Alus are “primate-specific” by definition (Batzer and Deininger 1991, 2002; Perna et al. 1992; Stoneking et al. 1997; Deininger 2011), it is worth noting that about one-fifth of nonprimate genome databases are contaminated with human sequences (Longo et al. 2011; Kryukov and Imanishi 2016). Therefore, unchecked in silico analyses of even high-quality databases, like UCSC or Ensembl, can generate spurious lineage-specific results, as we report in the supplementary table 1, Supplementary Material online, as an example about Alu sequences.

**Fig. 3.**
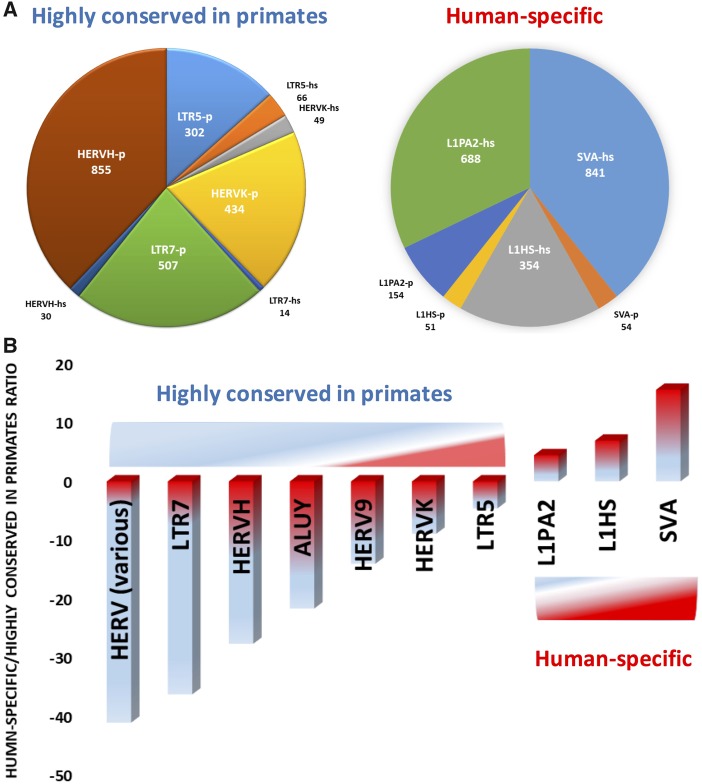
Evolutionary dynamics of highly conserved-in-primates and human-specific TE loci transcriptionally active in DLPFC of human brain. DLPFC-expressed TEs having > 99% of individual loci represented by primate-specific sequences (table 2 and supplementary tables 1–4, Supplementary Material online) were identified and analyzed for expression of primate- and human-specific TEs. TE loci expressing the higher numbers of human-specific versus highly conserved-in-primate transcripts and vice versa were identified and analyzed in detail. Note that all TE loci that express the largest numbers of molecularly distinct human-specific TEs in human DLPFC display both common and distinct features of the evolutionary histories as represented by both highly conserved-in-primates and human-specific sequences. (*A*) The number of distinct TE loci expressing the largest numbers of human-specific TEs in human DLPFC are shown. All identified TE loci represented by markedly distinct numbers are human-specific and highly conserved-in-primate sequences. (*B*) TE loci that express the largest numbers of molecularly distinct human-specific TEs in human DLPFC display distinct evolutionary dynamics and show markedly distinct ratios of human-specific to highly conserved-in-primate sequences.

With such a caveat in mind, we analyzed in detail all primate-specific TEs expressed in the human DLPFC (table 1 and supplementary tables 1–4, Supplementary Material online) to assess the representation of human-specific and highly conserved in primates TE loci. In our analyses, TE loci that have at least 95% of sequence identity during the direct and reciprocal conversions to the genomes of *H. sapiens* (hg38), Chimpanzee (*Pan Troglodytes*, v5), and Bonobo (*Pan paniscus*) were defined as highly conserved in primates (see Materials and Methods). Among DLPFC-expressed TEs having > 99% of individual loci represented by primate-specific sequences (table 3, fig. 3, and supplementary tables 1–4, Supplementary Material online), we identified 3,497 L1Hs, L1PA2, and SVA loci, the majority of which show a human-specific (1,883 loci = 53.8%) rather than primate-specific (259 loci = 7.1%) sequence identity pattern, with a 7.3 human-to-primate sequence identity ratio. An opposite pattern is characterizing the DLPFC-expressed LTR/HERV elements: in this case, the primates-specific to human-specific TE sequence identity ratio is higher in favor of a larger number of primate-specific than human-specific TEs. Of the 2,946 expressed HERVK, LTR5, HERV9, HERVH, and LTR7 loci that we have observed in our sample, 2,238 loci (76%) present at least a 95% sequence conservation across Chimpanzee, Bonobo, and *H. sapiens*, while only 169 loci (5.7%) encode human-specific transcripts (table 3 and fig. 3). Supplementary tables 1–4, Supplementary Material online, report the proportion of primate-specific, highly conserved-in-primates, and candidate human-specific elements for the various TE classes and families whose expression was quantified in human DLPFC samples.

We also carried out an extensive manual curation of the 4,687 human-specific expressed TE sequences in DLPFC and found that 51 over 1,240 L1Hs and 54 over 3,317 SVA transcripts sequences are also present in the genomes of Chimpanzee and Bonobo, representing *bona fide* primate sequences that are not human contaminated and thus supporting the hypothesis that TE sequences other than LTRs’ could have high levels of orthologous sequence conservation in primates (Jacques et al. 2013).

Our analysis based on DLPFC-TE transcriptome data seems highly congruent with the hypothesis that many incremental, independent and TE-associated regulatory changes rather than one singular phenotype-defining event occurred in the human brains during the evolution of human lineage to facilitate the emergence of our unique human brain functions. Intriguingly, a conceptually similar hypothesis has been formulated for protein-coding genes (Sousa et al. 2017). To further appraise this hypothesis, we considered a set of eleven genes previously identified as genetic elements with firmly established neurodevelopmental functions and well-documented genetic/genomic/epigenetic alterations of potential functional significance acquired within the human lineage after the divergence of humans and chimpanzees: *FOXP2, CNTNAP2, SRGAP2, ARHGAP11B, NPAS3, MEF2A, AUTS2, DYRK1A, NRG3, FOXP1, MEF2C* (table 4;Sousa et al. 2017). Remarkably, all these eleven genes are marked with TE loci that are transcriptionally active in the human DLPFC (table 4), 87.5–99.2% of which represent primate-specific sequences, while only about half of these genes present with human-specific TE loci. While a detailed analysis of the evolutionary pattern of these genes is beyond the scope of the present work, it is nonetheless interesting to note that the human-specific characteristics of *SRGAP2, ARHGAP11B, MEF2C*, *DYRK1A*, and probably *FOXP1* implicate a complex pattern of complete or partial gene duplication with or without copy number variations (Sudmant et al. 2010; Florio et al. 2015, 2016; Fossati et al. 2016; Bellmaine et al. 2017). These observations suggest that, although TEs cannot be considered the only mechanisms driving the evolution of the human brain, a large set of identified TE transcripts expressed in the human DLPFC and highly conserved during ∼8 My of primates’ evolution, are likely conveying important evolutionary-conserved and primate-specific regulatory functions.

**Table 4.** Examples of Genes Tagged by TE Transcripts in Human DLPFC with Already Established Neurodevelopmental Functions and Documented Genetic/Genomic/Epigenetic Alterations of Potential Functional Significance in the Human Lineage After the Divergence of Humans and Chimpanzees.

Gene names and Classification Categories	Functionally Relevant Features on the Human Lineage	TE Transcripts, *n*	Primate-Specific TE Transcripts, *n* (%)	Highly Conserved in Primates TE Transcripts, *n* (%)	Human-Specific TE Transcripts, *n*	Human-Specific TE Loci
FOXP2	Amino-acid substitutionsRegulatory sequence	151	115 (76.2 %)	144 (95.4%)	2	L1PA2
CNTNAP2	DNA methylation	1,323	1,035 (78.2 %)	1,224 (92.5%)	22	L1PA2; AluY; SVA; L1Hs
SRGAP2	Duplications	460	277 (60.2%)	420 (91.3%)	0	NA
ARHGAP11B	Duplications	28	26 (92.9%)	26 (92.9%)	0	NA
NPAS3	Highest density of human accelerated regions	347	172 (49.6%)	333 (96%)	1	L1PA2
MEF2A	Excess of SNPs in an upstream gene-regulatory region	124	101 (81.5%)	123 (99.2%)	0	NA
AUTS2	Regions of selective sweep in Modern Humans after the divergence with Neanderthals	460	367 (79.8 %)	427 (92.8%)	5	L1Hs
DYRK1A	Regions of selective sweep in Modern Humans after the divergence with Neanderthals	77	61 (79.2 %)	75 (97.4%)	0	NA
NRG3	Regions of selective sweep in Modern Humans after the divergence with Neanderthals	770	542 (70.4 %)	721 (93.6%)	1	AluSc
FOXP1	Functionally relevant protein–protein binding with the FOXP2	128	78 (60.9%)	112 (87.5%)	0	NA
MEF2C	Duplications, partial deletions, microdeletions and mutations linked with haploinsufficiency	286	193 (67.5%)	282 (98.6%)	0	NA

Note.—The Identification of primate-specific, highly conserved in primates, and human-specific TE sequences was performed as described in Materials and Methods.

NA, not applicable; detailed descriptions of specific genes and the list of primary references can be found in (Glinsky 2016, 2018; Sousa et al. 2017); TE transcripts, numbers of transcripts we have detected in our DLPFC samples.

### Exploring the Impact of TE Transcriptome Analysis on Investigations of Schizophrenia Pathogenesis

**Fig. 4.**
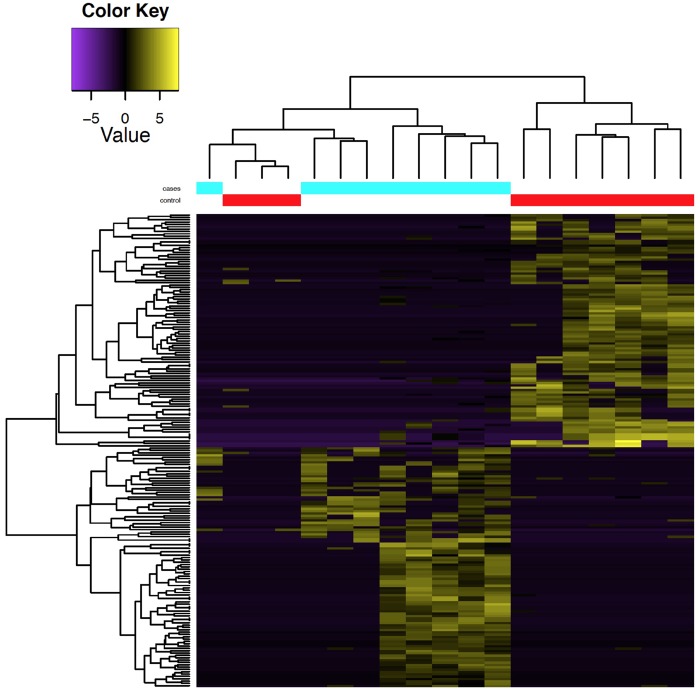
TE transcriptome in the DLPFC of Schizophrenia patients. The heatmap presents the pattern of the 112 up- and down-regulated TEs with Fold Change ± 4 comparing schizophrenic patients with controls. The vertical tree shows the distribution of schizophrenic patients (blue line) and controls (red line). The horizontal tree shows the distribution of the 112 expressed TEs and the colors in the body of the graph show which TEs are over- (yellow) or underexpressed (purple). The list of the up and down-regulated TEs used to build the graph are reported in supplementary table 5, Supplementary Material online.

To estimate the potential impact of lineage-specific TEs, we also looked at TE transcripts associated with schizophrenia. Given our very small sample size, we restricted our analyses to TE transcripts that we reliably detected in at least 50% of our samples (*n* = 114,172). We identified 1,689 differentially expressed transcripts with more than a 2-fold change and with a nominal significance threshold *P* value < 0.05 (fig. 4). About 88% of these differentially expressed TE transcripts (*n* = 1,484) mapped to 1,137 annotated genes, including 908 transcripts mapping to protein-coding genes, 191 to open reading frames (ORFs) of noncoding RNAs, and 38 to pseudogenes. The remaining 205 differentially expressed transcripts (12.1%) mapped to gene desert regions. We also found that 1,313 TE transcripts associated with schizophrenia are primate-specific (88.5%) and 39 human-specific (2.6%), supporting the hypothesis that most of the schizophrenia-associated TE transcripts appear originated from highly conserved sequences.

To explore the potential of TEs as biomarkers of SZ, we selected TEs that were preferentially expressed in at least 50% of cases or controls from the set of differentially expressed TEs (*P* < 0.05 and logFC > 2). We identified 203 TEs divided in 103 up- and 100 down-regulated (green in the heatmap) in cases compared with controls (supplementary table 5, Supplementary Material online). Selecting only the top 62 up-regulated (log fold-change >4) and the bottom 50 down-regulated (log fold-change <−4) TE transcripts, we found that we can classify cases and controls with a similar efficiency.

These schizophrenia-associated TEs are not randomly distributed across the genome, but they map with significant enrichment within 50 Topological Associated Domains (TADs) that are rapidly evolving in humans (Glinsky G 2015; Sexton and Cavalli 2015; Bonev and Cavalli 2016; Neems et al. 2016; Beagrie and Pombo 2017; Nagano et al. 2017). We noted that genes mapped with Schizophrenia-associated DLPFC-expressed TEs and located within these TADs (table 5) often manifest a clearly discernable pattern of brain-specific expression and many of these genes have been previously identified as possible candidates in Schizophrenia and/or other human brain disorders.

**Table 5.** Enrichment Analysis of Genes Tagged by TE Transcripts Differentially Expressed in DLPFC of Schizophrenia Patients and Mapped Within the Boundaries of 50 Rapidly Evolving in Humans Topologically Associating Domains (revTADs).

Classification Category	All Genes	Protein-Coding	Long ncRNAs, Including rRNAs	Small ncRNAs	Pseudogenes	Miscellaneous RNAs
Human genome	57,173	20,412	14,727	5,221	14,600	2,213
Mapped by TE transcripts in DLPFC within revTADs	1,408	731	555	5	104	14
Schizophrenia GES associated with TE transcripts in DLPFC	1,137	908	190	0	38	1
Schizophrenia GES located within revTADs	67	48	12	0	7	0
Percent of Schizophrenia GES located within revTADs	6	5	6	0	18	0
*P* value*	5.16E-11	0.0018	0.028	1	7.78E-09	0.99

TE, transposable elements; DLPFC, dorsolateral prefrontal cortex; PFC, prefrontal cortex; revTADs, rapidly evolving in humans topologically associating domains; GES, gene expression signature; ncRNAs, noncoding RNAs; rRNAs, ribosomal RNAs.

* *P* values were estimated using the hypergeometric distribution test.

## Discussion

We developed and implemented a comprehensive set of experimental and analytical approaches to unambiguously identify discrete TEs in postmortem samples of the human DLPFC. Starting with a genome-wide RNA-sequencing analysis, we identified 654,665 individual TE transcripts and mapped them with high confidence to 477,507 unique reference TE loci annotated in the human genome. We observed that the vast majority of TE transcripts unequivocally identified in human DLPFC mostly represent conserved primate-specific regulatory loci harboring TFBSs of three master transcription factors essential for human embryonic development and pluripotency maintenance in stem cells. The identification of TE loci with at least a 95% sequence conservation across Chimpanzee, Bonobo, and *H. sapiens*, argue in favor of their biologically relevant functions in the brains of Great Apes and potential multifaceted impacts on human brain physiology and pathology. Consistent with this hypothesis, we report the results of successful proof-of-principle analyses looking at association of expressed TEs with Schizophrenia, which also show that most associated TEs are primate-specific and putatively regulate genes in human-specific TADs. Our findings are consistent with the hypothesis previously introduced by others that altered expression of TEs in human DLPFC may affect the expression of protein-coding genes leading to malfunction of genetic regulatory networks during development as well as during the clinical manifestation of Schizophrenia and other brain disorders in humans (Chen et al. 2016; Guffanti et al. 2016; Mills et al. 2016; McConnell et al. 2017). Overall, our results support the hypothesis that many incremental independent genomic regulatory changes taking place over extended evolutionary periods, rather than one singular phenotype-defining event, have accumulated in the human brains during the speciation and evolution of human lineage to facilitate the emergence of our uniquely human brain functions. Although TEs cannot be considered the only mechanisms driving the evolution of the human brain, our findings suggest that a large set of identified TE transcripts expressed in the human DLPFC and highly conserved during ∼8 My of primates’ evolution, are likely conveying important evolutionary-conserved and primate-specific regulatory functions.

Our results rely on three critical elements of novelty in the study of TE expression in human tissues. First, we adopted a total RNA library preparation (NuGEN, see also Materials and Methods) that overcomes most of the limitations characteristics of the more commonly used Trueseq library preparation. For example, Trueseq is known to be prone to introduce errors induced by contaminant genomic DNA (Boivin et al. 2018; Carrell et al. 2018). NuGEN offers unique advantages as it allows for high sensitivity to genomic DNA, as also supported by the experimental validation tests we performed in our study, in addition to ribo-depletion, which is essential for noncoding RNA detection. Recently other RNA-sequencing approaches have been released that show higher sensitivity than Trueseq. A promising tool for future analyses is the RNA-seq workflow TGIRT, which leverages a processive reverse transcriptase isolated from a thermostable group II intron, that reveals higher fidelity than conventional reverse transcriptases to map a diverse population of transcripts, including small noncoding RNAs (Nottingham et al. 2016; Wu and Lambowitz 2017). Second, other computational methods and software exist that use RNA-seq data to quantify the expression of TEs, like TEtranscript (Jin et al. 2015) or SalmonTE (Jeong et al. 2018). However, these approaches are specifically designed to measure the expression levels of entire subfamilies of TEs, while our novel method is capable of quantifying the expression of single TE loci at their specific genomic location. Third, we provided the most extensive and thorough independent experimental validation of our in silico detected TE expression profiles to date.

While our overall findings represent the best validated results from a preliminary investigation on the possible role of expressed TEs in the human brain, they surely need confirmation in larger samples. However, a few considerations are possible even considering the limited power of our approach. The impressively large number of transcriptionally active TEs observed in a well-defined brain region supports the hypothesis that the regulatory genome was indeed essential in shaping the evolutionary mechanisms that define the structural and functional organization of the human brain (Konopka et al. 2009, 2012; Konopka and Geschwind 2010; Somel et al. 2011, 2013; Burbano et al. 2012; Liu et al. 2012; Maricic et al. 2013; Marnetto et al. 2014, 2018; Boyd et al. 2015). This contribution to the human brain evolution from the noncoding genome seems at least equal in relevance and importance to the already well-defined findings derived by the analyses of gene variants, either nucleotide substitutions or Copy Number Variants, which implicate such genes in specific evolutionary and developmental steps of brain configurations, from increase in brain size to cortical folding. Future analyses that will access larger samples will probably identify even more expressed TEs than those we have reliably found, but our prediction is that new findings will not significantly affect the large prevalence of primate-specific expressed elements, and will probably help to better characterize those elements that appear to be human-specific.

Another interesting point is that TEs that are expressed in the human DLPFC do not appear to be randomly distributed across the myriads annotated TE loci of the human genome, but they look constrained within defined TADs and seem mostly active by inserting a limited number of TFBSs. These expressed TEs maintain many characteristics that have been originally found in hESC and human primordial germ cells (hPCG). While most of the putative transcription factor-binding sites that are active in hESC are essentially silenced in differentiated cells, NANOG-binding sites embedded within primate and human-specific LINE (long interspersed nuclear elements) and LTR (long terminal repeats) sequences seem to be still transcriptionally active in the human brain. This pattern suggests that specific TE-derived regulatory elements in the neural genome maintain early developmental characteristics along the life span and that probably some functional gene-networks in the human brain are organized similarly to early human developmental and germ cell programs. If confirmed, then this is another characteristic that would distinguish the neural from other somatically differentiated tissues.

Much remains to be done, other than confirmatory analyses in larger samples, most notably identifying the specific functional role of expressed TEs and which gene network they putatively control for. While an RNA-sequencing technology together with a comparative genomic approach is a critical step to pinpoint potentially functional elements, their biological relevance must then be studied with other methods and techniques. The importance of this issue is highlighted by recent experimental approaches (Whalen et al. 2016; Cao et al. 2017; Hait et al. 2018) that aims at establishing physiological maps of common and cell type-specific putative regulatory elements as pioneered by the Roadmap Epigenomics consortium (Kundaje et al. 2015). The initial emerging indications suggest that up to 50–70% of the predicted enhancers-promoter (E–P) links involve an intronic enhancer, since probably most of the E–P interactions are occurring within TADs, while at least 30% of enhancers are not fitting the widely adopted assumption that links enhancers to their nearest gene (Hait et al. 2018). At present, indeed, our current knowledge is still limited by the relative scarcity of studies investigating tissue and cell specific expression of putative regulatory elements. To facilitate these future experiments, we have been able to unequivocally map a large number of TE-derived candidate regulatory loci to their specific chromosomal locations, identify hundreds of thousands of novel RNA molecules expressed in human DLPFC, reliably quantify their expression and test whether their expression is altered in human brains affected by pathological conditions. Our work demonstrates the benefits of detailed systematic explorations of high-precision genome-wide maps of TE-derived transcriptomes in defined anatomical regions of the human brain to reveal exciting and readily available fundamental and translational opportunities for the immediate future.

## Materials and Methods

### Samples

Total RNA from the Dorso-Lateral Prefrontal Cortex (DLPFC—Brodmann area 46) of nine schizophrenia cases and ten psychiatrically healthy controls was obtained from the UCI Brain Bank. Donors or their first-degree relatives signed an informed consent to the UCI Brain Bank to have their tissues donated for scientific research, under an UCI-IRB approved protocol. Our sample includes 6 women and 14 men, whose ages at death ranged from 31 to 68 (average = 46.1 ± 11.4 [of which CTRLs: 48 ± 13, SZ: 44.3 ± 10, *P* = ns]). Brain tissues have been collected within a mean postmortem interval (PMI) of 19 ± 4 h. All specimens presented an RNA Integrity Number (RIN) ranging between 6.3 and 9.1 (average 7.9 ± 0.7) and a pH from 6.0 to 7.1 (average 6.4 ± 0.3). To control for the presence of other potential disease states, we conducted neuropathological examinations and ruled out neurofibrillary tangles, senile plaques or Lewy bodies in our samples. Following dissection, samples were flash frozen. Total RNA was extracted from 80 to 100 mg of frozen tissue using the Qiagen mRNA kit. RNA concentration was assessed using a NanoDrop spectrophotometer and RNA integrity using an Agilent 2100 Bioanalyzer RNA Nano Chip. Cases and controls were matched for gender and age.

### Library Preparation

RNA-seq is usually carried out using polyadenylated (PolyA) tail selection. Noncoding RNA transcripts, though, may or may not have PolyA tails, which makes PolyA selection not appropriate for our study. To address this limitation, we decided to use the NuGEN Encore Complete Library preparation protocol that does not rely on PolyA selection. From 100 ng of total RNA, the kit enriches for non-rRNA in NGS libraries during cDNA synthesis. The first strand cDNA synthesis is carried out using proprietary primers to create double-stranded cDNA, which retains RNA strand information. No dedicated steps are required to reduce rRNA levels. The resulting cDNA is converted to NGS libraries using reagents and adaptors provided in the same kit. The Encore Complete RNA-Seq Library Systems have been designed for strand-specific expression analysis by incorporation of a nucleotide analogue during the second strand cDNA synthesis, and subsequent ligation, to a pair of double-stranded adaptors also containing the same analogue in one strand. After ligation, the cDNA strand and adaptor containing the analogue are selectively removed (Strand Selection), leaving only one cDNA strand, with both adaptor sequences attached. The Encore Complete RNA-Seq Multiplex Systems provide optional barcoding to further optimize efficiencies and cost savings in transcriptome sequencing. This product is then converted into a sequence-ready library by PCR amplification.

### Paired-End Sequencing

We sequenced our samples on an Illumina Hi-Seq Analyzer 2500 at the UCI Genomic High Throughput Core Facility. We optimized multiplex libraries on a single flow cell to reach a minimum of 60–70 million reads per subject using 100 cycles of paired end sequencing to detect also low abundance transcripts and obtained between 68 and 109 M reads per subjects representing >40-fold enrichment for target sequences. Paired-end (PE) RNA-Seq raw reads were binned according to the barcodes and the barcodes and adaptors were trimmed away and finally saved in FASTQ format files containing sequences plus quality information in Phred format.

### Sequencing Quality Control Assessment

The preprocessed reads were then subjected to quality control using FastQC and reads were filtered out if mean quality falls <20 (Andrews 2010).

### Genome-Guided De Novo Assembly by Trinity

Genome-Guided de novo assembly (GGDNA) is a method offered by Trinity to perform de novo transcriptome assembly at each locus leveraging prior alignment of reads to the genome partitioned according to annotated loci, via an available reference genome annotation to define these loci (Grabherr et al. 2011; Haas et al. 2013). The genome is only being used as a substrate for grouping overlapping reads into clusters that were then separately fed into Trinity for de novo transcriptome assembly. This approach is particularly appropriate for TE mapping as the sequences of TEs are highly repetitive and therefore represent greatly similar sequences that might be shared by multiple loci across the genome. This may lead to potential alignment of the same reads to multiple copies of the same TE. Single TE loci can be distinguished one from another by leveraging the emergence of single nucleotide variations or INDELs within their sequence since the time of TE’s original insertion in the human genome. So, for example, two TEs of the same class and family, for example, HERVKC4, might be present at two different genomic loci and still share a great proportion of their sequences, but the variations accumulated within their sequence over time since the original insertion are different making each sequence unique. We reasoned that this method allows partitioning reads to locus prior to doing any de novo assembly, thus improving their alignment to specific loci and decreasing the chance to be aligned multiple times to different loci. The first step of GGDNA consists in the alignment of reads to the TE reference genome which is provided by Repbase/Repeatmasker database for the human genome version hg38. The quality filtered reads were aligned to TE sequences annotated in the Repbase/Repeatmasker database to provide the initial partition of the reads according to TE reference sequences (Smit 2013–2015), using HISAT2 (Kim et al. 2015; Pertea et al. 2016). The second step consists in the identification and assembly of TE transcripts, including the assignment of their strand specificity. TE transcript sequences are saved in a Fasta file, which represents the de novo assembly of TE transcriptome. The GGDNA application to TE transcriptome assembly is schematically presented in figure 1.

### Quality Control of Alignment of Assembled Transcripts

First, we used Megablast to align each assembled transcript to the reference set of TE sequences deposited in RepBase and accessed through Repeatmasker. For each transcript, we calculated the proportion of the sequence that is successfully aligned to a reference TE locus. We filtered out transcripts with <95% of identical matches with the reference sequence of the TE to which it was aligned and only transcripts that align at least 90% with a reference TE were retained.

### Quantification of Expression Levels

We used Kallisto (v.0.43.0) to quantify the level of expression for each transcript, which allows both reads correctly matched or not with their mate to be accounted for in the quantification procedure (Bray et al. 2016). Kallisto uses a reference transcriptome index to quantify reads at their correct location: we used the sequences of TEs deposited in RepBase and accessed through Repeatmasker as reference transcriptome to generate an index. Transcript per million (TPM) values were then calculated using Kallisto with default parameters for all RNAseq samples from DLPFC.

### Differential Expression

We used the EdgeR Bioconductor package to test for differential expression between cases and controls (Robinson et al. 2010; McCarthy et al. 2012). EdgeR is set to keep only those transcripts that have at least one read per million in at least two samples. To define the signature of up- and down-regulation signature of TE expression in DLPFC, we stratified the set of differentially expressed TEs (nominal *P* < 0.05) into transcripts expressed in at least 50% of cases (up-regulation) and controls (down-regulation).

### Quantitative RT-PCR

To validate the identification of active TE loci, RNA of four samples was used for TE-specific RT-PCR analyses. Primers were designed in regions of selected TEs (supplementary table 6, Supplementary Material online) with amplicon sequences unique to the location of the specific TE loci. To ensure species specificity and that primers were univocally mapping TE amplicons at their specific genomic location, all primer sets and corresponding TE amplicons were aligned using BLAST against the reference genomes of *Homo sapiens* and *Rattus norvegicus*. The required target specificity was defined by the 100% identity with no gaps along the entire length of the amplicon (supplementary table 6, Supplementary Material online). The vast majority of reference TEs (89.5%) selected for the experimental validation were identified by different segment fragments as result of splicing of the nascent RNA of the reference TE. It should be noted that the amplicons generated for the validation were selected to uniquely map locus-specific sequences and were not specifically designed to span a splice junction. The results of these experiments validated one of the key features of bioinformatics pipeline enabling the identification of specific TE loci transcribed in the human DLPFC. Real-time PCR Sybr green primers utilized in validation experiments are described in supplementary table 6, Supplementary Material online. About 500 ng of DNA was used per reaction. Reactions were quantified using Applied Biosystems StepOnePlus Real-Time PCR system. All samples were run in triplicate. Both Ct value and melting temperature represent the mean of each triplicate.

### cDNA Preparation

RNA samples were quantified using Qubit. RNA samples were reversed transcribed according to the manufacturer’s protocol using 1 µg of RNA as template (QuantiTect Reverse Transcription Kit, Qiagen). The –RT sample (pooled, with equal parts of all samples; i.e., 250 ng per sample) was prepared similarly except that in place of the addition of the reverse transcription enzyme, the sample was instead treated with RNAse A solution (Qiagen). Per manufacturer’s protocol, reverse transcription is prefaced with genomic wipeout with DNAse treatment. Therefore, the –RT sample represents residual genomic content following gDNA digestion.

### Rat gDNA Preparation

gDNA was isolated from a whole frontal cortex sample from a Sprague Dawley rat. Briefly, tissue was homogenized with lysis buffer (100 mM Tris–Cl; 50 mM EDTA; 1% SDS(w.v.); pH8.0) before proteinase K digestion and RNAse treatment (Invitrogen; Qiagen, respectively). gDNA was then isolated by phenol–chloroform extraction and prepared via ethanol precipitation. gDNA was then reconstituted, concentration and quality measured via Nanodrop 2000c spectrophotometer (ThermoScientific) before diluting to 100 ng/µL.

### Visualization of RT-qPCR Products

RT-PCR products from human samples were pooled for each target. Pooled PCR products were then prepared by phenol–chloroform extraction followed by ethanol precipitation. Reconstituted products were then measured via Nanodrop before running out on an agarose gel. Products were prepared for gel electrophoresis with orange dye loading buffer. A 100-bp ladder was similarly prepared. Samples were run for 2 h at 120 V on a 2% agarose (w.v) gel. After running, the gel was stained with ethidium bromide diluted in TAE and washed with fresh TAE. The gel was then imaged using the Bio-rad ChemiDoc XRS+ imaging system following UV light activation.

### Definitions of Primate-Specific, Highly Conserved-in-Primates, and Candidate Human-Specific TE Loci

Identification of 1) primate-specific, 2) highly conserved-in-primates, and 3) human-specific TE loci among those that are transcriptionally active in human DLPFC was performed as previously described (Glinsky 2015; Glinsky 2016). In brief, TE loci that have failed the liftover conversion from the human genome (hg38) to the mouse genome (mm10) at a minimum remapping rate of 10% (10% of bases) were defined as 1) primate-specific loci; TE loci that have at least 95% of bases remapped (the minimum ratio of bases that must remap is 0.95:1) during the direct and reciprocal conversions to the genomes of *H. sapiens* (hg38), Chimpanzee (*Pan Troglodytes*, v5), and Bonobo (*Pan paniscus*) were defined as 2) highly conserved in primates; TE loci that have failed to remap at the minimum ratio of 10% bases during the conversion from the *H. sapiens* genome to both Chimpanzee (*Pan Troglodytes*, v5) and Bonobo (*Pan paniscus*) genomes were defined as 3) candidate human-specific loci.

## Supplementary Material

Supplementary Data
