# Supplementary material for: Novel Bioinformatics Approach Identifies Transcriptional Profiles of Lineage-Specific Transposable Elements at Distinct Loci in the Human Dorsolateral Prefrontal Cortex
Source: Mol Biol Evol. 2018 Jul 20;35(10):2435–53. doi: 10.1093/molbev/msy143 (PMC6188555; doi:10.1093/molbev/msy143)
Supplement: Supplementary Data [file msy143_supp.zip › Suppl Table 8.docx]

**Supplemental Table 8. RT-qPCR melting temperatures of selected *de novo* transcript amplicons.** TE transcripts were selected from across families of class I TEs. 18S ribosomal RNA and GAPDH were included as internal controls. As shown, all subjects were run for 40 cycles for each target. In addition, no template control (NTC), a negative reverse transcription sample (-RT) and a rat genomic DNA sample (Rat gDNA) were also run in tandem. 18S primers were built against the 18S hyperconserved domain, both primers matching exactly with both hg38 and rn6 reference sequences, and served as a positive control for all samples. Conversely, GAPDH primers were built against hg38 reference sequence and contained two mismatches per primer against *rattus norvegicus* rn6 reference sequence. multiple, indicates multiple melting temperatures indicating multiple products.

|  | Melt Temp. (°C) |  |  |  |  |  |
| --- | --- | --- | --- | --- | --- | --- |
| Target | G1-1817 | G2-2035 | G3-2353 | G4-2410 | -RT | Rat gDNA |
| 18S | 81.63859558 | 81.64154561 | 81.44252014 | 81.59475962 | 81.2428716 | 81.35136414 |
| GAPDH | 80.75035858 | 80.60385132 | 80.59189606 | 80.7461853 | undeter. | 79.2583313 |
| AluJo | multiple | multiple | multiple | multiple | multiple | multiple |
| AluSX | 80.59213257 | 80.1553421 | 80.29295349 | 80.44719696 | multiple | multiple |
| L1HS, chr2, set 1 | 76.0063324 | 75.85929871 | 75.84873199 | 76.00136566 | 75.85447693 | 75.85929871 |
| L1HS, chr2, set 2 | 77.79640198 | 77.5004425 | 77.49887085 | 77.64224243 | 77.49557495 | 77.79724884 |
| L1PA2, set 1 | 77.4980545 | 77.35124969 | 77.34053802 | 77.34390259 | 77.34638214 | 77.64963531 |
| L1PA2, set 2 | 76.0063324 | 75.85929871 | 75.84873199 | 75.85219574 | 76.0063324 | 77.64963531 |
| L1M1, set 1 | 74.01055145 | 74.020401 | 73.71903229 | 73.86514282 | 73.86106873 | 78.50540161 |
| L1M1, set 2 | 75.20639801 | 75.06690216 | 75.05604553 | 75.05931854 | 56.0728302 | multiple |
| L1PA7, set 1 | 75.95380402 | 75.66490173 | 75.8035202 | 75.95620728 | 75.8087616 | 75.36589813 |
| L1PA7, set 2 | 73.71159363 | 73.57189941 | 73.56109619 | 73.71398926 | 73.71563721 | 73.87090302 |
| L1HS | 76.55973816 | 76.41693115 | 76.40855408 | 76.41664124 | 76.41030121 | 78.95731354 |
| SVA_B | 78.95071411 | 78.8092804 | 78.79908752 | 78.95342255 | 78.95071411 | multiple |
| LTR5_HS, chr3 | 78.95071411 | 78.80788422 | 78.94850159 | 78.95342255 | multiple | multiple |
| SVA_D | 84.1809845 | 84.18842316 | 84.17779541 | 84.18339539 | 84.33042145 | multiple |
| HERVK, chr8, set 1 | 75.87162781 | 75.7325592 | 75.72246552 | 75.87643433 | 72.5899353 | 76.92475891 |
| HERVK, chr8, set 2 | 72.5899353 | 72.45115662 | 72.44120026 | 72.74382019 | 72.5899353 | multiple |
| HERVH, set 1 | 75.57329559 | 75.58340454 | 75.57331848 | 75.57813263 | 75.42412567 | multiple |
| HERVH, set 2 | 78.10914612 | 77.82072449 | 77.95968628 | 77.96455383 | 77.81080627 | 78.26818848 |
| LTR5_HS, chr9 | 79.45631409 | 79.16169739 | 79.29606628 | 79.15563965 | 79.30149841 | multiple |
