# Supplementary figures and images for: Novel Bioinformatics Approach Identifies Transcriptional Profiles of Lineage-Specific Transposable Elements at Distinct Loci in the Human Dorsolateral Prefrontal Cortex
Source: Mol Biol Evol. 2018 Jul 20;35(10):2435–53. doi: 10.1093/molbev/msy143 (PMC6188555; doi:10.1093/molbev/msy143)

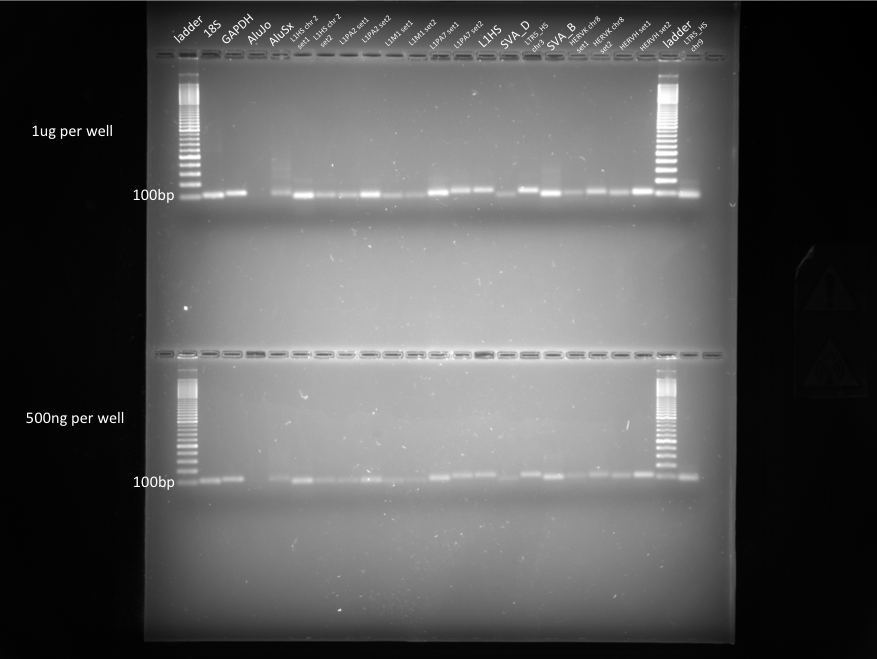

Supplement: Supplementary Data [file msy143_supp.zip › SupplFigure1.png]
